# Novel Carboxymethyl Cellulose/Gelatin-Based Film Incorporated with Zein-Stabilized Lemon Essential Oil Pickering Emulsion for the Preservation of Cherries

**DOI:** 10.3390/foods13162602

**Published:** 2024-08-20

**Authors:** Kaiwen He, Wenyang Sheng, Li Yang, Yicheng Yang, Tingting Tang, Chenzhi Wang, Guangyang Jiang, Yongqiang Tian

**Affiliations:** 1 College of Biomass Science and Engineering, Sichuan University, No. 24 South Section 1, Yihuan Road, Chengdu 610065, China; kevin22493369@163.com (K.H.); 13162336616@163.com (W.S.); ylsc1398@163.com (L.Y.); yycscuer@163.com (Y.Y.); yqtian@scu.edu.cn (Y.T.); 2 Key Laboratory of Leather Chemistry and Engineering, Sichuan University, Ministry of Education, Chengdu 610065, China; 3 College of Agriculture and Forestry Science and Technology, Chongqing Three Gorges Vocational College, Chongqing 404160, China; cqsxtangtingting@163.com; 4 Institute of Agro-Products Processing Science and Technology, Sichuan Academy of Agricultural Sciences, Chengdu 610066, China

**Keywords:** composite film, zein, lemon essential oil, pickering emulsion, fruit preservation

## Abstract

In this study, a zein-stabilized lemon essential oil Pickering emulsion (ZLPE) was incorporated into a carboxymethyl cellulose/gelatin (CMC/GL) composite film to develop a bio-based packaging material with bioactive properties. The average droplet size of the ZLPE was measured at 3.62 ± 0.08 μm, with a zeta potential of −31.33 ± 0.32 mV, highlighting its excellent stability. The image results of confocal laser microscopy and scanning electron microscopy validated the uniform distribution of ZLPE in the film. The incorporation of ZLPE reduced the water solubility of films by 45.90% and decreased its water vapor permeability by 22.61%, thereby enhancing its hydrophobicity. Additionally, the ZLPE-loaded film improved mechanical properties, enhanced UV-blocking capabilities, and increased thermal stability. The introduction of ZLPE led to the antioxidant activity of the CMC/GL film increasing by six times the original level and endowed it with outstanding antibacterial properties. As a result, cherries packaged with the ZLPE film demonstrated superior preservation performance and extended shelf life in the preservation experiment, exhibiting the film’s potential as a food packaging material.

## 1. Introduction

Due to their excellent properties and affordability, petroleum-based plastics are extensively used in the packaging industry [1]. However, the non-biodegradability of petroleum-based polymers has resulted in severe environmental pollution, necessitating the identification of viable substitutes [2]. Bio-based polymers have garnered significant attention owing to their environmentally friendly properties and biodegradability. Currently, extensively studied bio-based polymers mainly include polysaccharides and proteins. These materials are not only biodegradable but also exhibit good biocompatibility and film-forming properties, making them potential replacements for plastics [3]. Carboxymethyl cellulose (CMC) is a widely used food additive, commonly employed as a thickener, stabilizer, and emulsifier. Its excellent film-forming capability was attributed to its high molecular weight and polymer structure [4]. However, pure CMC films are limited in flexibility. Enhancing their mechanical properties through functional complementation with other matrices is an effective approach [5]. Gelatin (GL), a product of incomplete protein hydrolysis, possesses good biocompatibility and film-forming ability. Previous studies have shown that CMC/GL composite films demonstrate better mechanical properties and lower water vapor permeability compared to pure CMC films [6]. However, the insufficient antioxidant and antibacterial activities of CMC/GL films remain a crucial factor restricting their application.

Plant essential oils are safe plant extracts extensively used in food packaging films [7]. Lemon essential oil (LEO), a natural extract obtained from the peel of lemon fruit, can serve as a substitute for synthetic preservatives in food due to its antioxidant properties and antibacterial activity [8]. However, the direct addition of LEO to food may alter the food’s original flavor due to its potent aroma [9]. Incorporating LEO into packaging films preserves the antioxidant and antibacterial effects of LEO without compromising the flavor profile of food, thereby extending shelf life. However, the volatile nature of essential oils and their limited dispersibility in film-forming solutions pose challenges to maintaining their biological activity in films over extended periods [10]. More specifically, it is challenging for essential oils to disperse uniformly as small droplets within the film, hindering their ability to exert biological activities stably over time. To address this issue, essential oils can be encapsulated within a medium to enhance their stability and prolong their biological activity [11].

Pickering emulsions, stabilized by solid particles, have garnered attention for their superior stability in comparison to traditional surfactant-stabilized emulsions [12]. Consequently, Pickering emulsion systems are extensively utilized in food packaging films for encapsulating essential oils and thereby ensuring their long-term stability. In recent years, natural organic solid particles such as protein particles have been chosen to stabilize Pickering emulsions on account of their safety and biocompatibility [13]. Specifically, protein particles stabilized Pickering emulsions possessed enhanced colloidal stability as the protein particles formed a viscoelastic interfacial film at the interface of the two phases. This interfacial film generates steric and electrostatic hindrances, inhibiting the coalescence of dispersed droplets [14]. Zein, a water-insoluble protein abundant in hydrophobic amino acids, is widely employed in the manufacture of food coatings and films on account of its degradability and safety [15]. In a previous study, it was observed that the chitosan film incorporated with zein-stabilized Pickering emulsions containing clove essential oil demonstrated excellent hydrophobicity, antioxidant activity, and antibacterial activity [16]. Furthermore, the addition of zein-gallic acid conjugates stabilized Pickering emulsions and resulted in films exhibiting slow-release behavior, enhanced antioxidant activity, and increased antibacterial properties [17]. Thus, the construction of Pickering emulsion systems is considered an efficient method for encapsulating essential oils, which improves the stability and increases the bioactivity of the packaging films.

In this study, to improve the dispersion uniformity and stability of lemon essential oil, ZLPE was prepared and incorporated into the CMC/GL film-forming solution to produce bioactive packaging films. The effects of ZLPE concentrations on the composite films’ microstructure, morphology, mechanical properties, optical characteristics, hydrophobicity, and thermal stability were investigated using instruments and techniques such as SEM, XRD, and FT-IR. On this basis, the antioxidant properties, antibacterial activity, and cherry preservation effects of the ZLPE-loaded film were further explored, aiming to demonstrate the potential of this composite film containing ZLPE in food packaging applications. This encapsulation technology enables an effective and sustainable strategy for constructing smart films in the packaging field.

## 2. Materials and Methods

### 2.1. Materials

CMC sodium salt was purchased from Chengdu Kelong Reagent Co., Ltd. (Chengdu, China). GL was obtained from Sangon Biotech Co. Ltd. (Shanghai, China). Zein and D-(+)-gluconic acid δ-lactone (GDL) were acquired from Shanghai Aladdin Biochemical Technology Co. Ltd. Lemon essential oil (LEO) was sourced from Huashuo Spice Oil Co., Ltd. (Jian, China). All other reagents were of analytical grade. *Staphylococcus aureus* (ATCC29213) and *Escherichia coli* (ATCC25922) were supplied by the China Industrial Culture Collection Center (Beijing, China).

### 2.2. Preparation of ZLPE

The zein nanoparticles (ZNPs) were prepared according to the method of Sun et al., with slight modifications [18]. Zein (1.5 g) was dispersed in 50 mL of deionized water, and the pH of the solution was adjusted to 12.5 by adding 0.5 mol/L NaOH solution. The mixture was stirred on a magnetic stirrer for 30 min until the zein dissolved completely. Next, 1.55 g of GDL was added and stirred for 2 h to adjust the pH to 8.0. Subsequently, the dispersion of ZNP and LEO were mixed in a 1:1 volume ratio and homogenized for 10 min to form the ZLPE.

### 2.3. Characterization of ZLPE

#### 2.3.1. Droplet Size Distribution and Zeta Potential

The droplet size distribution and zeta potential of ZLPE were determined utilizing a nanoparticle sizer (NanoBrook Omni, Brookhaven, GA, USA). Prior to conducting the measurements, the ZLPE was diluted by a factor of 100.

#### 2.3.2. Confocal Laser Scanning Microscopy (CLSM)

The microstructure of ZLPE was observed using a confocal laser scanning microscope (STELLARIS 5, Leica Microsystems Inc., Wetzlar, Germany). The protein and oil phases within the ZLPE were labeled with fluorescein isothiocyanate and perylene staining, respectively. To evaluate the dispersity of ZLPE in film-forming solutions, ZLPE was dispersed in CMC/GL solutions at concentrations of 0.25%, 0.5%, and 1.0%.

### 2.4. Fabrication of Composite Films

**Figure 1:**
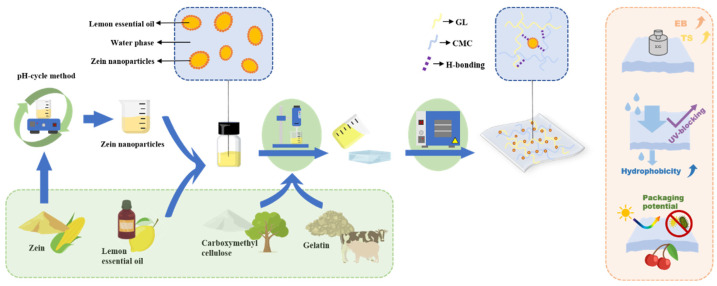
Schematic illustration of the preparation process of the ZLPE-loaded films.

The preparation process of the film-forming solution is illustrated in Figure 1. The solution was prepared by dispersing 4.0 g CMC and 2.0 g GL into 300 mL deionized water, followed by stirring on a magnetic stirrer at 60 ℃ until all solutes were dissolved. The glycerol (0.4%, *w*/*w*) was then incorporated into the fully dissolved solution, followed by the addition of ZLPE in varying proportions (0%, 0.25%, 0.5%, 1.0% *v*/*v*). The solution was homogenized for a duration of 10 min, and subsequently sonicated and degassed for 30 min using a vacuum pump. Next, 25 g of the processed film-forming solution was poured into an acrylic mold and dried at 45 ℃ for 10 h. The resulting films were labeled as ZL-0%, ZL-0.25%, ZL-0.5%, and ZL-1.0% according to the respective concentration of the ZLPE. 

### 2.5. Rheological Properties

The rheological properties of film-forming solutions were analyzed utilizing a rheometer (MCR302, Anton Paar, Ostfildern, Germany) equipped with 25 mm parallel plates. Flow curves were generated across a spectrum of shear rates varying from 0.1 to 1000 s^−1^ at 25 °C.

### 2.6. Structure and Morphology

#### 2.6.1. Scanning Electron Microscope (SEM)

To observe the cross-section and morphology of the films, they were fixed on aluminum plates, gold-sputter coated, and then observed through a scanning electron microscope (Apreo 2s, Thermo Scientific, Waltham, MA, USA) at an accelerating voltage set to 5.00 kV.

#### 2.6.2. X-ray Diffraction (XRD)

The XRD patterns were obtained using an XRD diffractometer (Ultima IV, Rigaku, Tokyo, Japan). The films were scanned in the 5–85° angular range with a speed of 10°/min.

#### 2.6.3. Fourier Transform Infrared (FT-IR)

An infrared spectrometer (Spectrum 3, PerkinElmer, Waltham, MA, USA) was employed to analyze the film spectra within the spectral region of 600–4000 cm^−1^ at a spectral resolution of 4 cm^−1^. Each film underwent 32 scans using attenuated total reflection (ATR).

### 2.7. Mechanical Properties

#### 2.7.1. Thickness

The digital micrometer (San Liang, Dongguan, China) was utilized to measure the thickness of the films, achieving an accuracy of 0.001 mm. Ten randomly selected points on every film were measured to calculate the average thickness.

#### 2.7.2. Tensile Strength (TS) and Elongation at Break (EB)

TS and EB of films were examined employing a universal testing machine (Instron Co., Norwood, MA, USA). Prior to assessing the mechanical properties, the films were cut into 2 cm × 5 cm rectangles, and an initial distance of 2.5 cm was established for the grips.

### 2.8. Optical Properties

#### 2.8.1. Appearance and Color

The exterior images of the films were captured by a camera (EOS R50, Canon, Tokyo, Japan). The external lighting conditions and camera parameters remained constant throughout the shooting process. A colorimeter (CR-400, Konica Minolta, Osaka City, Japan) was employed to assess the color characteristics of the film surface. The total color difference index (∆*E*) was calculated from the measured *L* (lightness), *a* (redness/greenness), and *b* (blueness/yellowness) parameters using the following Equation (1):

(1) $$\Delta E=\sqrt{{\left(L-L^{*}\right)}^{2}+{\left(a-a^{*}\right)}^{2}{+(b-b^{*})}^{2}}$$

where L*, a*, and b* were the color parameters of the white standard plate (L* = 91.61, a* = −0.06, b* = 2.43).

#### 2.8.2. Transmittance

A UV-visible spectrophotometer (LAMBDA 1050, PerkinElmer, Waltham, MA, USA) was utilized to determine the absorbance characteristics of the film within the wavelength range of 280 nm to 800 nm, and the corresponding transmittance was calculated using the following Equation (2):

(2) $$Transmittance\left(\%\right)=\frac{1}{10^{A}}\times 100$$

where *A* was the absorbance of the film.

### 2.9. Water-Resistance Properties

#### 2.9.1. Moisture Content (MC) and Water Solubility (WS)

The squares obtained from the films measured 2 cm × 2 cm in size and were subsequently weighed (m_0_). Then, they were subjected to drying at 105 °C for 12 h to ensure complete evaporation of the moisture, followed by weighing (m_1_). Next, the films were immersed in 15 mL of water for a duration of 12 h. The remaining insoluble portions were filtered, and then dried for 12 h at a temperature of 105 °C, and their weight was determined (m_2_). The MC and WS of the films were calculated according to Equations (3) and (4):

(3) $$MC\left(\%\right)=\frac{m_{0}-m_{1}}{m_{0}}\times 100$$

(4) $$WS\left(\%\right)=\frac{m_{1}-m_{2}}{m_{1}}\times 100$$

#### 2.9.2. Water Vapor Permeability (WVP)

The WVP of the films was assessed following previously reported methodologies [19]. To begin, 5 g of dried calcium chloride was added into a 25 mL conical flask and the flask was sealed with film. The flasks were subsequently placed in a desiccator maintained at 20 °C and 50% relative humidity and weighed every 8 h until 72 h. The WVP of the films was determined using Equation (5):

(5) $$WVP=\frac{W\times D}{t\times A\times ∆P}$$

where *W* was the increased weight in the conical flask (g), *D* was the film thickness (m), *t* was the duration (s), *A* was the permeation area (m^2^), and Δ*P* was the vapor pressure between the pure water and dry atmosphere (2339 Pa). 

#### 2.9.3. Water Contact Angle (WCA)

A contact angle microscope (HKCA-40, HARKE, Beijing, China) was used to obtain the WCA of the films with the sessile drop method. The films were cut into rectangular shapes measuring 1 cm × 5 cm and affixed onto a slide, and distilled water was dropped at three random locations.

### 2.10. Thermogravimetric Analysis (TGA)

A thermal analyzer (TG209F1, Netzsch, Waldkraiburg, Germany) was utilized to evaluate the thermal stabilities of the films over a temperature range from 35 °C to 800 °C, with a heat-up rate of 10 °C per minute, in a nitrogen environment.

### 2.11. Antioxidant Activity 

The antioxidant capabilities were evaluated through the determination of their capacity to scavenge the 2,2-diphenyl-1-picrylhydrazyl (DPPH) radicals. The experimental method was based on previously reported methods and underwent minor modifications [20]. In brief, 50 mg samples were excised from films with varying ZLPE contents and added to 10 mL of DPPH solution (0.2 mmol/L, 50% *v*/*v* methanol). Subsequently, the mixed solution was placed in a dark setting and incubated for 1 h. Ultimately, the absorbance of the solution was measured at 517 nm. The determination of DPPH radical scavenging capacity was conducted in accordance with Equation (6):

(6) $$DPPH\;radical\;scavenging\;percentage\;\left(\%\right)=(\frac{A_{0}-A_{1}}{A_{0}})\times 100$$

where A_1_ was the absorbance of the film-forming solution containing different additive amounts of ZLPE mixed with DPPH solution and A_0_ was the absorbance of the DPPH solution as the control group.

### 2.12. Antibacterial Activity

In the experiment, *E. coli* and *S. aureus* were selected as representatives of Gram-negative bacteria and Gram-positive bacteria, respectively. Circular slices with a diameter of 6 mm were cut out from the films. Subsequently, these slices were placed onto LB media that were pre-inoculated with either *E. coli* or *S. aureus* bacteria. Following an incubation period of 12 h at 37 °C, measurements of the diameter of the inhibition zone were conducted.

### 2.13. Application for Cherry Preservation

Fresh cherries with similar shapes and sizes were picked and randomly categorized into six groups. After cleaning, one group was designated as the control group without undergoing any treatment. The other group was wrapped with commercially available polyethylene (PE) film. Finally, the remaining four groups were packaged using ZLPE films of different concentrations. The cherry fruits were maintained at 25 ± 1 °C for a period of several days. The freshness of cherries was assessed based on their appearance, weight loss rate, firmness, and soluble solids content.

#### 2.13.1. Appearance of Cherries

The camera (EOS R50, Canon, Tokyo, Japan) was used to record variations in the appearance of the cherries, and all the photos were taken under the same light conditions and camera parameters. To prevent the films from interfering with the observation of cherries, the films covering the cherries were removed prior to each recording. After shooting, the cherries were re-wrapped in the films.

#### 2.13.2. Weight Loss Rate of Cherries

At the initiation of the preservation experiment, the cherries were weighed (m_0_). Subsequently, after a storage period, the cherries were reweighed (m_t_). The weight loss rate was calculated using Equation (7):

(7) $$Weight\;loss\;rate\left(\%\right)=\frac{m_{0}-m_{t}}{m_{0}}\times 100$$

#### 2.13.3. Firmness of Cherries

The texture analyzer (TA. XT plus, Stable Micro System, Godalming, UK) was employed to assess the firmness of the cherries. A needle probe was used to pierce vertically into the cherry to a depth of 6 mm at a rate of 1 mm/s.

#### 2.13.4. Soluble Solids Content of Cherries

The soluble solids content of cherries was measured using a refractometer (SW-35S, SWEVY, Guangzhou, China). The cherry juice used in the measurement was obtained by crushing the sample cherry and then subjecting it to filtration.

### 2.14. Statistical Analysis

In this study, a minimum of three replicates were conducted for each trial. The statistical data were analyzed using SPSS Statistics 26 (IBM, New York, NY, USA). The significance of differences was evaluated through Duncan’s multiple tests (*p* < 0.05).

## 3. Results and Discussion

### 3.1. Characterization of ZLPE

**Figure 2:**
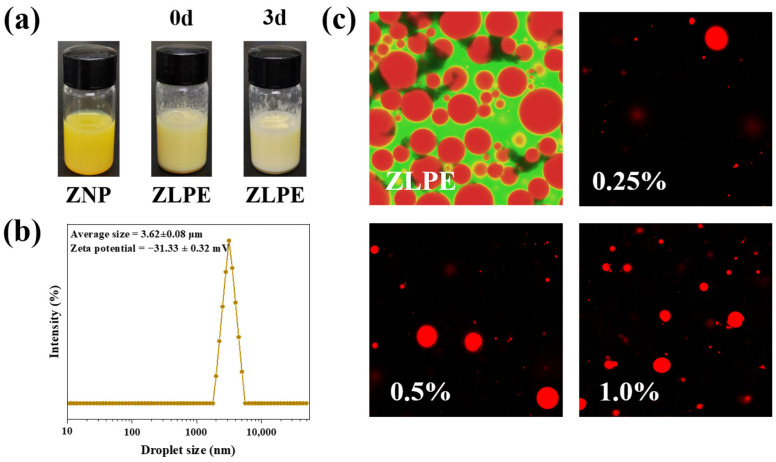
Appearance of ZNP, freshly prepared ZLPE and stored ZLPE for 3 days (**a**), droplet size distribution of ZLPE (**b**), the CLSM images of ZLPE itself and ZLPE dispersed in the film-forming solution (**c**).

The illustration in Figure 2a indicated that ZNP suspensions appeared yellow, while ZLPE appeared opalescent. ZLPE did not stratify after being stored for 3 d. The dimensions of droplet size play a vital role in determining the stability of emulsions. As depicted in Figure 2b, the ZLPE displayed an average droplet size of 3.62 ± 0.08 μm with a unimodal distribution. Compared to the average particle size of zein/soluble soybean polysaccharide nanoparticle-stabilized Pickering emulsions, the particle size of the ZLPE in this study was smaller [21]. The variations in the preparation methods of the two ZNPs might account for this difference. In this study, the pH-cycle method was employed to fabricate ZNPs, and the incorporated GDL was capable of reducing the pH of the solution uniformly and gradually, rather than instantaneously like hydrochloric acid. Thus, the size of the fabricated ZNPs is smaller and the size distribution is more uniform [18]. Generally speaking, a zeta potential absolute value exceeding 30 mV indicates a stable colloidal system, as increased electrostatic repulsion between particles prevents emulsion droplet coalescence [20]. In our research, the ZLPE exhibited a zeta potential of −31.33 ± 0.32 mV, indicating robust stability of the system. ZLPE exhibits prolonged storage stability without stratification due to its small droplet size and high absolute value of zeta potential, which positively influences its long-term stable biological activity in films.

The morphology of ZLPE and the distribution of ZLPE within the film-forming solution were illustrated in Figure 2c. The ZLPE group represented the appearance of dyed pure emulsion, while the 0.25%, 0.5%, and 1.0% groups represented the appearance of dyed emulsion proportionally dispersed in CMC/GL solution. The oil phase was dyed red, and the protein-containing water phase was dyed green. The ZLPE droplets were spherical, plump, and intact, suggesting their stability. The ZLPE image depicted dispersed red droplets within a greenish background, indicating an oil-in-water (O/W) nature. This observation was similar to one previously noted by Yang et al. [20]. Moreover, ZLPE at various concentrations demonstrated favorable distribution in the CMC/GL solution. The high-viscosity solution could prevent the aggregation of emulsion droplets, thus aiding in the uniform distribution of ZLPE within the film [22].

### 3.2. Characterization of the Composite Films

#### 3.2.1. Rheological Properties of the Film-Forming Solutions

**Figure 3:**
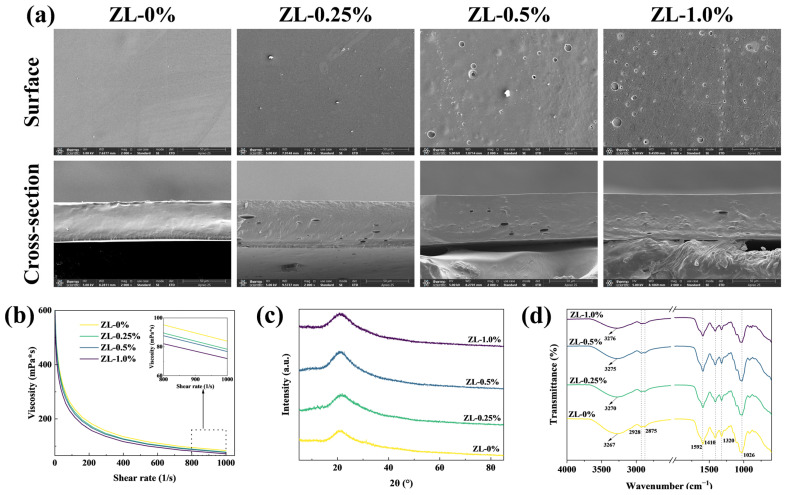
SEM images of films (**a**). The rheological curves of the film-forming solutions (**b**). XRD patterns (**c**), and FT-IR patterns (**d**) of films.

Rheological properties are instrumental in assessing the influence of ZLPE on the film microstructure [23]. Based on the rheological curve depicted in Figure 3b, the viscosity of the solutions decreased with the enhancement of the shear rate, suggesting the presence of non-Newtonian properties in the fluid. This non-Newtonian fluid can be sprayed from a nozzle, which then coats the exterior of an item and creating a film on the surface, thereby broadening the application scope of film-forming solutions [24]. A minor reduction in viscosity was detected in the film-forming solutions when ZLPE was added. The reduction in viscosity may be attributed to the incorporation of ZLPE, which causes the concentration of CMC/GL within the film-forming solution to decrease, thereby reducing the thickening capacity of CMC/GL [25]. However, the high viscosity of the pure CMC/GL film could hinder film formation, and the decreased viscosity resulting from the incorporation of ZLPE might be advantageous for the formation of the films [26]. Yu et al. found in previous research that CMC/GL-based film-forming solutions exhibited similar rheological properties [27].

#### 3.2.2. Morphology and Structure

The impact of ZLPE on the microstructure of the films can be assessed through SEM images. Microstructure images of films containing different concentrations of ZLPE were depicted in Figure 3a. The ZL-0% film exhibited a flat and consistent surface, while its cross-section was continuous and dense. Compared to the ZL-0% film, the surface of the films incorporating ZLPE became rougher and exhibited some hole structures, with the number of holes increasing with the ZLPE concentration. These holey structures likely result from the movement of emulsion droplets towards the surface of the film as the film-forming solutions were dried and their volatiles evaporated [28]. On the cross-section, evenly distributed elliptical pores were observed. This phenomenon may be due to the coagulation and flocculation of emulsion droplets, which could cause the emulsion molecules to merge into bigger droplets within the films and ultimately give rise to the distinctive pore morphology [17]. The holey structures observed on the surface and cross-section of the film are consistent with those reported by Bangar et al. [29], who identified that incorporating clove bud essential oil in a Pickering emulsion led to the formation of microporous structures in both the surface and cross-section of pearl millet starch films. The uniformly distributed pore structures demonstrate the effective integration of ZLPE within the film matrix, as well as the enhanced dispersibility of ZLPE. In addition, these pore structures may facilitate the release of bioactive substances, thereby enhancing the bioactivity of the films [30].

XRD patterns can be used to determine the crystalline nature of samples [27]. The XRD patterns of all films, from ZL-0% to ZL-1.0%, were shown in Figure 3c. The addition of ZLPE to CMC/GL films had little effect on the position of diffraction peaks, only altering the intensity of the absorption peak. From ZL-0% to ZL-0.5%, progressively enhanced diffraction peaks were observed, which could be associated with the intensified intermolecular forces such as hydrogen bonds between ZLPE and the matrix of the film [26]. The intensity of the diffraction peak for the ZL-1.0% film was actually lower than that observed in the ZL-0.5% film, possibly due to the impact of discontinuous distribution of numerous hydrophobic substances within the film on the hydrogen bonding network structure. Zhao et al. also found similar changes in the XRD pattern after adding Pickering emulsion to the corn starch/cassia gum composite film [31]. These results demonstrate that ZLPE is capable of forming stronger hydrogen bonds and other intermolecular interactions with the film matrix. However, an elevated level of ZLPE might alter the inherent structure of the film, potentially reducing the mechanical properties.

The FT-IR spectra are useful for characterizing the chemical structure and various molecular interactions in the films. Figure 3d illustrated the FT-IR spectra of composite films loaded with varying ZLPE concentrations. Compared to the ZL-0% film, the ZLPE-loaded films did not exhibit any new absorption peaks in the absorption spectrum, meaning that no chemical reactions occurred between the ZLPE and other components. The absorption peaks at 2928 cm^−1^ and 2875 cm^−1^ were related to the asymmetric and symmetric stretching vibrations of -CH_2_- and -CH_3_, respectively [32]. The peak at 1592 cm^−1^ is caused by the symmetric and asymmetric carboxyl groups in CMC [33]. The bands at 1410, 1320, and 1026 cm^−1^ corresponded to the -OH, C-H, and C-O bonds in the CMC polysaccharide structure, respectively [32]. The broad peak at 3267–3276 cm^−1^ was associated with the stretching vibration of free O-H groups [34]. The addition of ZLPE flattened the absorption peak at 3267–3276 cm^−1^ and increased the wave number. This might result from the robust hydrogen bonding formation between ZLPE and the matrix, reducing the amount of free -OH in the film [26]. The observed results of newly formed hydrogen bonds were consistent with XRD results. Based on the analysis of XRD and FT-IR data, it is concluded that there is no chemical reaction between ZLPE and CMC/GL films. Furthermore, ZLPE has little impact on the overall structure of the films. The interaction between ZLPE and the film matrix primarily occurs through intermolecular forces, such as hydrogen bonding between zein and CMC/GL. These hydrogen bonds exert a certain influence on the physical properties of films.

#### 3.2.3. Optical Properties

**Figure 4:**
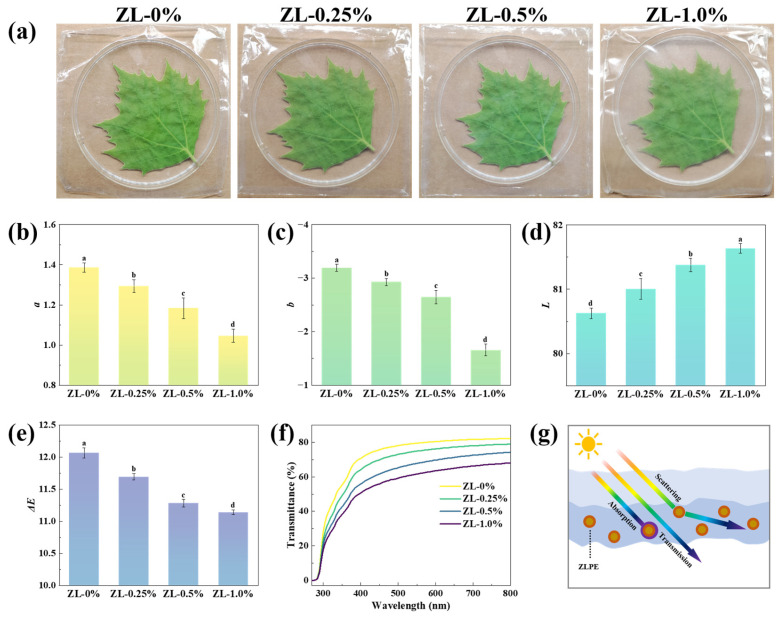
Appearance (**a**), *a* (**b**), *b* (**c**), *L* (**d**), and Δ*E* (**e**) of films. UV/visible light absorption spectrum (**f**) and schematic diagram of light-blocking principle (**g**). a–d: Different lowercase letters indicate significant difference between data (*p* < 0.05).

The color and transparency of the films have a certain impact on people’s perception of the state of the packaged items [35]. The appearance and color difference data of the films were shown in Figure 4. In terms of appearance, all films were initially almost colorless and transparent. However, with the increase of ZLPE content, the films gradually turned white, and their transparency decreased. This phenomenon was consistent with the decrease in light transmittance of the films within the visible region as shown in Figure 4f, which occurred as the addition of ZLPE increased. This effect was particularly pronounced at an additional level of 1.0%. The reduced transparency of the film may be attributed to the distribution of ZLPE droplets within the film, causing light scattering as it passes through. Moreover, an increase in film thickness could also lead to a decrease in film transparency. The *a* and *b* absolute values in the ZL-0% group were the highest and the *L* value was the lowest. With the addition of ZLPE, the *a* value gradually decreased from 1.39 ± 0.02 to 1.05 ± 0.03 and the *b* value increased from −3.19 ± 0.06 to −1.66 ± 0.11, revealing that the red and blue colors of the films gradually weaken. The *L* value experienced a progressive enhancement, rising from 80.63 ± 0.08 to 81.64 ± 0.08, which suggests a concomitant increase in the luminosity of the films. Consequently, it could be calculated that the Δ*E* value decreased from 12.07 ± 0.08 to 11.14 ± 0.03, reflecting a reduction in color difference.

The UV-blocking performance constitutes a crucial element in ascertaining the effectiveness of food packaging films, owing to the destruction of nutrients in food caused by UV radiation [36]. As shown in Figure 4f, the transmittance of the films decreased with increasing amounts of ZLPE. ZLPE-loaded films exhibited lower transmittance in both visible and ultraviolet spectra, which shows an enhanced UV/visible light barrier effect. The enhanced UV-blocking performance of the film may be attributed to the intense absorption of UV light by LEO [37]. Additionally, the absorption of UV light by ZNP and the light scattering effect caused by the emulsion droplets distributed within the film also contributed to the decreased transmittance [17]. The result was consistent with the literature, which reported that the transmittance of chitosan/gelatin film decreased with the incorporation of cinnamon essential oil Pickering emulsion [38]. In summary, dispersed Pickering emulsion drops can diminish light transmittance, while the ZLPE-loaded film possesses outstanding UV-blocking properties, effectively safeguarding the nutritional content of food.

#### 3.2.4. Mechanical Properties

**Figure 5:**
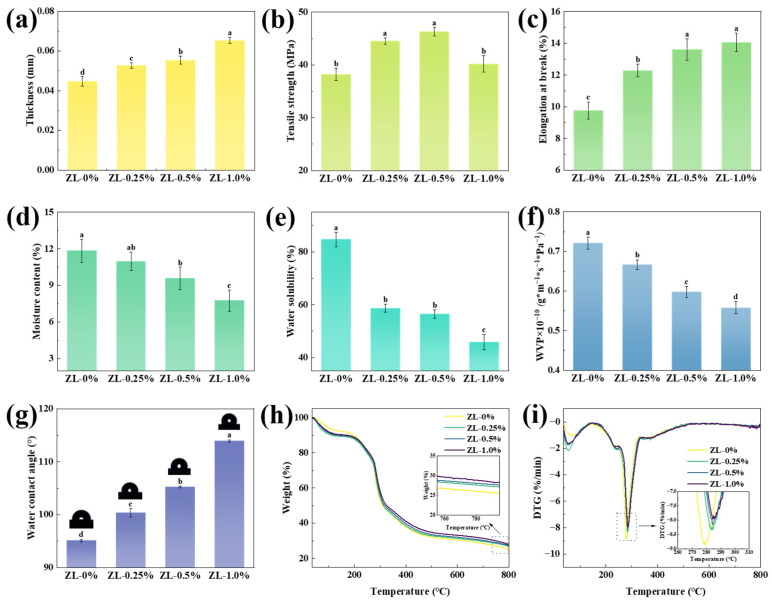
Thickness (**a**), TS (**b**), EB (**c**), MC (**d**), WS (**e**), WVP (**f**), and WCA (**g**) of ZL-0%, ZL-0.25%, ZL-0.5% and ZL-1.0% films. TG (**h**) and DTG (**i**) curves of films. a–d: Different lowercase letters indicate significant difference between data (*p* < 0.05).

Figure 5a illustrates the thickness of both the ZL-0% film and the ZLPE-loaded films. The ZL-0% film displayed a measurement of 0.045 ± 0.003 mm in thickness, while the ZL-1.0% film exhibited a recorded thickness of 0.065 ± 0.002 mm, which was a significant increase of approximately 46% (*p* < 0.05). This result was likely relative to the incorporation of ZLPE, increasing the solids content in the films [10]. This observation was further supported by the SEM images of the films.

TS and EB represent the strength and flexibility of films, respectively, determining their ability to maintain integrity in practical applications [39]. The measured mechanical property data are presented in Figure 5. With the rise of ZLPE concentration from 0 to 0.5%, TS increased from 38.2 MPa to 46.3 MPa. However, TS decreased to 40.2 MPa when the concentration of the ZLPE further increased to 1.0%. According to the XRD and FT-IR results, hydrogen bonds formed between ZLPE and the film matrix. Therefore, it can be inferred that the increase in TS at low ZLPE concentrations was likely ascribed to hydrogen bond interactions between the biopolymer matrix within the film and the emulsion [40]. At higher ZLPE concentrations, the altered film structure significantly impacted mechanical properties. As discerned in the SEM cross-section image presented above, the pore structure within the film originated from the high concentration-induced clustering of emulsion droplets, which might exert a more significant influence on the TS of the film [17]. Similarly, the XRD pattern showed the highest diffraction peak intensity in the ZL-0.5% film, which decreased in the ZL-1.0% film, confirming that high ZLPE concentrations disrupted the film’s original hydrogen bonding network structure, affecting both the XRD peak and mechanical properties. Previously, Bangar et al. observed in their research that the TS of the films incorporated with cellulose nanocrystals stabilized clove bud oil Pickering emulsion also displayed the trend of initially increasing and subsequently decreasing [29]. Additionally, EB increased with higher ZLPE concentrations, ranging from 0–1.0%. The EB of the ZL-0% film was 9.76 ± 0.54%, whereas that of the ZL-0.25% film was 12.49 ± 0.40%. The addition of merely 0.25% ZLPE enhanced the EB of CMC/GL films by approximately 28%. When the concentration of ZLPE attained 1.0%, EB increased to 14.06 ± 0.59%. This might be due to ZLPE acting as a plasticizer, enhancing the film’s flexibility through the variability of oil droplets [41]. The interaction between ZLPE and the matrix of the film led to the development of new hydrogen bonds, which consequently modified the initial hydrogen bonding pattern and affected the EB [16]. Liu et al. reported similar trends in TS and EB of high internal phase Pickering emulsions loaded konjac glucose films [41].

#### 3.2.5. Water-Resistance Properties

Figure 5d,e demonstrate the MC and WS of the composite films, respectively. The MC of the ZL-0% film was 11.84 ± 0.94%, whereas that of the ZL-1.0% film was 7.75 ± 0.88%. The WS of the ZL-0% control film reached 84.71 ± 2.72%, consistent with other films of similar composition [42]. However, the addition of ZLPE resulted in a significant decrease in WS (*p* < 0.05), reaching a minimum of 45.83 ± 2.94% at 1.0% ZLPE concentration. The decrease of MC and WS might be attributed to the inclusion of hydrophobic LEO within ZLPE, which resulted in an enhanced hydrophobic nature of the films. Furthermore, the interaction between the film matrix and the emulsion via hydrogen bonds reduced the availability of hydrophilic groups on the matrix molecules, making it more difficult for the matrix to bind with water molecules, thereby decreasing the MC and WS [43]. The high WS of pure CMC/GL films is a significant limitation for their use as packaging materials. However, the addition of ZLPE greatly reduces their water solubility, making it possible to apply this novel composite film in the field of food packaging.

The WVP of the films, as presented in Figure 5f, displayed a reduction upon the incorporation of ZLPE. The WVP of the films in the ZL-0% group was 0.72 ± 0.01 × 10^−10^ g×m^−1^ × s^−1^ × Pa^−1^, whereas the WVP of the films in the ZL-1.0% group was 0.56 ± 0.02 × 10^−10^ g×m^−1^ × s^−1^ × Pa^−1^, representing a reduction of approximately 22%. The reduction in WVP can be ascribed to the homogeneous distribution of the ZLPE within the films, disrupting the continuous pathway for water molecules and increasing tortuosity [24]. Additionally, the increased film thickness elongated the pathway for water molecules, further impeding the passage of water. Almasi et al. observed a similar phenomenon, where the WVP of the fruit gum film was reduced by about 8-fold after the addition of the Marjoram essential oil Pickering emulsion [44]. The low WVP helps inhibit moisture movement between food items, packaging, and the ambient atmosphere. This prevention of moisture loss in fresh food maintains product integrity and enhances consumer visual appeal.

WCA is commonly utilized for the determination of the hydrophilicity or hydrophobicity of a material. It is commonly held that values more than 65° indicate hydrophobic properties and values less than 65° indicate hydrophilic properties [41]. A higher WCA signifies increased hydrophobicity. The WCA of the films containing varying concentrations of ZLPE are depicted in Figure 5g. The hydrophobic ZLPE significantly raised the WCA from 95.08° to 113.90° (*p* < 0.05). These results indicated that the untreated film was hydrophobic, and its hydrophobic nature was further enhanced upon the incorporation of the ZLPE. Relevant research on CMC/GL films corroborated this result [27]. The increase in WCA was mainly due to the addition of ZLPE, which enhanced the overall film’s hydrophobicity, and the hydrogen bonding between the ZLPE and the CMC/GL film matrix partially replaced intermolecular interactions between water molecules and the matrix [39]. Furthermore, the enhanced surface roughness of the film, as depicted in the SEM images, also contributed to the higher WCA value. The increased WCA, along with the results for MC, WS, and WVP, validated the enhanced hydrophobicity of the films due to the ZLPE addition, suggesting that the ZLPE-loaded film exhibits potential as a material for food packaging applications.

#### 3.2.6. Thermal Stabilities

Thermal stability is evaluated through analyzing the weight change with increasing temperature. The CMC/GL films demonstrated three stages of weight loss. During the first stage, occurring at 50–90 °C, the process was characterized by the removal of water from the film matrix through evaporation. The subsequent stage, which took place at 220–240 °C, was attributed to the volatilization of LEO and the breakdown of glycerin [28]. The main weight reduction occurred in the third stage, which was in correspondence with the thermal decomposition of film matrix [42]. According to the TG curve of Figure 5h, the residual mass increased as ZLPE was incorporated. As depicted in the DTG curve presented in Figure 5i, the maximum thermal degradation temperature of the film loaded with ZLPE exceeded that of the pure CMC/GL film, while the maximum thermal degradation rate of the film containing ZLPE was inferior to that of the pure CMC/GL film, suggesting an improved thermal stability through the incorporation of ZLPE. This enhancement was likely associated with the hydrogen bonding existing between ZLPE and the film matrix, and stronger hydrogen bonding would result in greater thermal stability of the films [40]. This was similar to the improvement in thermal stability of corn starch/cassia gum composite film by the addition of anise essential oil Pickering emulsion [30]. Food packaging films are generally not designed for use in high-temperature conditions. However, the increased thermal stability enhances the range of applications for the film.

### 3.3. Antioxidant Activity and Antibacterial Activity

**Figure 6:**
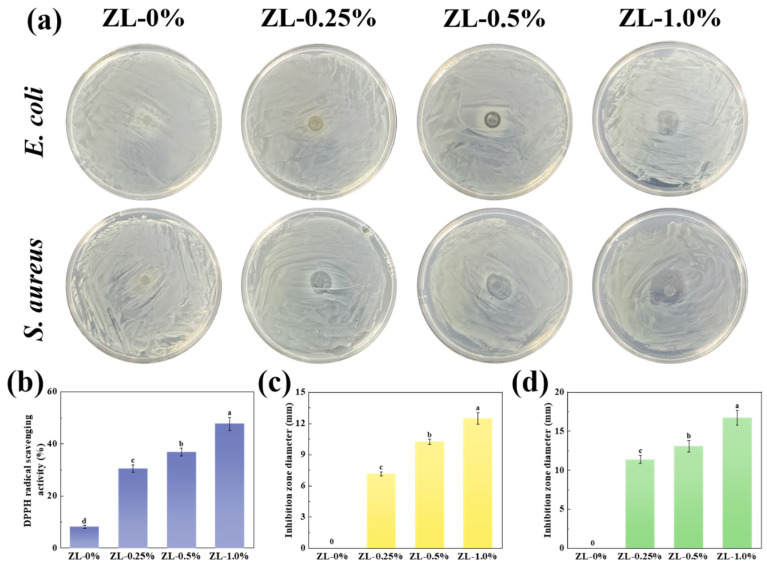
Antibacterial effect on *E. coli* and *S. aureus* (**a**). DPPH radical scavenging activity (**b**). Diameter of inhibition zone against *E. coli* (**c**) and *S. aureus* (**d**). a–d: Different lowercase letters indicate significant difference between data (*p* < 0.05).

Incorporating bioactive substances with antioxidant properties into packaging films is a crucial strategy to prevent food degradation due to oxidation and to extend shelf life [39]. The DPPH free radical scavenging activities of composite films are shown in Figure 6b. The DPPH free radical scavenging rate of the CMC/GL film was 8.17 ± 0.55%, showing a small antioxidant capacity. This might be a result of the amino acids and peptides in the gelatin providing electrons to pair with free radicals [45]. However, the inclusion of ZLPE led to an increase in the antioxidant activity, ultimately peaking at 47.70 ± 2.52%, primarily due to the presence of LEO [46]. LEO contains multiple active ingredients, including limonene, β-pinene, γ-terpenes, and aldehydes, which have strong antioxidant activities [47]. Furthermore, the antioxidant properties of zein might also contribute to the ZLPE-loaded film antioxidant activity [48]. These results demonstrated that the incorporation of ZLPE is an effective approach to augmenting the antioxidant capacity of composite films.

The antibacterial ability of the films was assessed quantitatively by measuring the size of the inhibition zone, wherein a greater diameter denotes more potent antibacterial properties. The inhibition zones against *E. coli* and *S. aureus* of films with varying ZLPE concentrations are illustrated in Figure 6a. The ZL-0% film exhibited no inhibitory zone, indicating the absence of antibacterial activity in CMC/GL. Similarly, the lack of inhibition zones on control CMC/GL films was also noted in a previous study [32]. When ZLPE was incorporated into the films, inhibition zones were detected, suggesting that the emulsion conferred antibacterial activity to the films. The diameter of the inhibition zone of the ZLPE-loaded films against *E. coli* and *S. aureus* are depicted in Figure 6c,d. The diameter of the inhibition zone for *E. coli* increased from 7.13 ± 0.21 mm to 12.50 ± 0.56 mm as the ZLPE dosage increased from 0.25% to 1.0%. Similarly, the diameter of the inhibition zone for *S. aureus* also increased from 11.37 ± 0.50 mm to 16.70 ± 0.95 mm. The antimicrobial activity of the film was correlated with the limonene in LEO, and the antibacterial ability was improved with the increase of limonene content. According to a previous study, limonene was capable of penetrating and destroying the cell membrane structure of bacteria, thereby killing the bacteria [49]. Furthermore, the film-forming solutions with the same ZLPE concentration exhibited different inhibition zone diameters against *E. coli* and *S. aureus*. This observation could be explained by the difference in cell wall composition between *E. coli* (Gram-negative bacterium) and *S. aureus* (Gram-positive bacterium). In Gram-positive bacteria, the cell wall teichoic acids facilitate the entry of essential oils into the bacterial cells. In contrast, the outer membrane of Gram-negative bacteria, which is mainly made up of lipopolysaccharides, hinders the permeation of hydrophobic essential oils [50].

### 3.4. Preservative Ability

#### 3.4.1. Appearance

**Figure 7:**
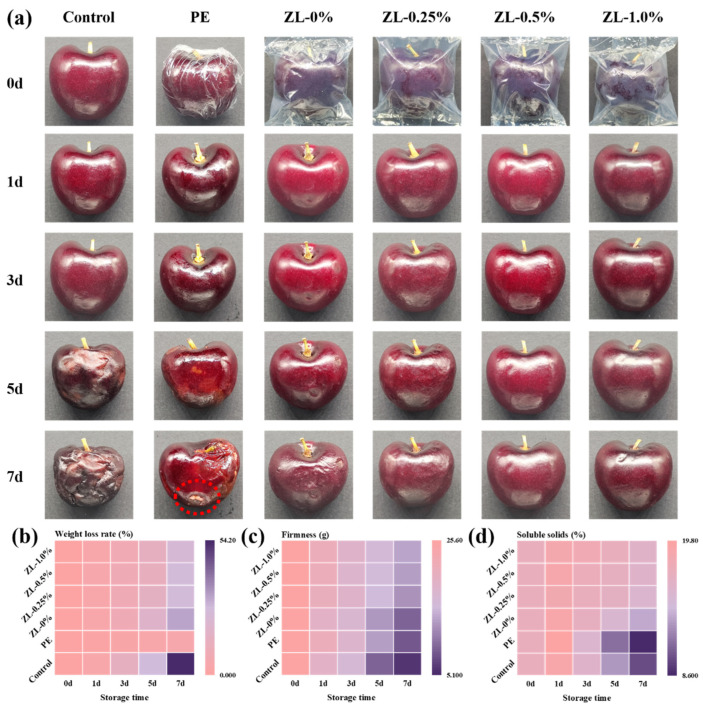
Preservation effect of films on cherries (**a**). The weight loss rate (**b**), firmness (**c**), and soluble solid content (**d**) of cherries.

The appearance and morphology of cherries treated with different methods over the storage period are shown in Figure 7a. Since the 5th day, there was obvious shrinkage on the surface of cherries in the control group. A partial white colony appeared on the surface of cherries in the PE group. Cherries in the CMC/GL film group also showed skin wrinkling on day 7, but to a lesser extent than in the control group. In contrast, cherries wrapped with composite films containing ZLPE maintained a relatively smooth surface and full shape. The wrinkling of cherries might be associated with softening and water evaporation during the ripening process in storage [51]. The film on the surface of the fruit functions as a protective layer, hindering water vapor exchange, and the antioxidant capacity provided by ZLPE to the film inhibits the ripening and softening of the cherry. 

During this period, it is noteworthy that only cherries in the PE group exhibited significant colony growth on the surface, accompanied by a putrid odor and the scent of alcohol following anaerobic fermentation. This phenomenon may be attributed to the limited gas exchange capability of the PE film, which creates a humid environment conducive to microbial growth [52]. In contrast, although the ZLPE film also boasts exceptional water vapor barrier properties, it additionally possesses certain hygroscopic qualities that help maintain dry cherry surfaces. Furthermore, the antioxidant and antibacterial properties conferred by the ZLPE film create an advantageous environment for prolonged fruit preservation. Cherries wrapped in ZLPE film retain their natural aroma without acquiring any hint of lemon essential oil scent; this could be attributed to the minimal amount of lemon essential oil incorporated into the film and its sustained-release effect subsequent to forming Pickering emulsion.

#### 3.4.2. Weight Loss Rate

As depicted in Figure 7b, the cherries in the control group exhibited the highest weight loss rate at 54.11 ± 2.47%, while the PE group was the lowest, merely 5.20 ± 0.13%. The weight loss rates of cherries wrapped with the films decreased as the concentration of ZLPE increased. The final weight loss rate for the ZL-0% group was 31.72 ± 2.05%, while the ZL-1.0% group had a rate of 23.87 ± 2.20%. The decrease in water content resulting from respiration and transpiration was primarily responsible for the reduction in fruit weight. The film reduces fruit transpiration by inhibiting the release of water vapor, indicating that a lower WVP of the film corresponded to a reduced rate of fruit weight loss [35]. PE films possess limited gas exchange capacity and are incapable of absorbing water; therefore, minimal weight change in cherries is expected in the PE group. The rate of weight loss in cherries packaged with the CMC/GL composite film was correlated with the WVP value of the film.

#### 3.4.3. Firmness

The firmness of the fruit is indicated by the strength of its skin. The firmness data of the fruits are presented in Figure 7c. All cherries exhibited varying degrees of decline in firmness, with the control group showing the greatest decrease from 25.5 ± 1.2 g to 6.1 ± 0.6 g. The firmness of cherries in the PE group decreased significantly on the 5th day (*p* < 0.05), and it was merely 8.5 ± 0.7 g on the 7th day. The cherries wrapped with the CMC/GL composite films exhibited a slower decrease in firmness compared to the control group, with the final firmness of the cherries in the ZL-0% group measuring 9.4 ± 0.7 g and that of the cherries in the ZL-1.0% group measuring 13.6 ± 0.6 g. During storage, the cell walls of the fruit were hydrolyzed by enzymes, resulting in a decrease in firmness [35]. The firmness of cherries in the PE group remained high during the initial 3 days; however, it decreased rapidly in the subsequent period, presumably because the moist environment within the PE film in the later stage caused the cherries to rot and soften. The films, particularly the one containing ZLPE, can proficiently delay the ripening of cherries and prevent the decrease in cherry firmness. These results suggested that the ZLPE-loaded film can effectively preserve the firmness of the fruit, thereby prolonging shelf life.

#### 3.4.4. Soluble Solid Content

Figure 7d showed that the soluble solid content of cherries followed a pattern of initial growth followed by decline. The concentration of water-soluble solids within a fruit is closely linked to its degree of ripeness, and an increase in this concentration may result from the breakdown of the fruit’s cell walls and the evaporation of water. Over some time, the soluble solid content of cherries decreased, likely due to the consumption of organic matter resulting from the respiration of the fruit [53]. Zeng et al. also documented a preliminary rise in the concentration of soluble solids within cherry films, which was followed by a subsequent decline [52]. On the 7th day, the soluble solids content was 10.1 ± 0.8% in the control group and 16.2 ± 0.8% in the ZL-1.0% group. However, the final soluble solid content in PE group was the lowest, only 8.7 ± 0.5%. The rapid decrease in the soluble solid content of cherries wrapped with PE films may be attributed to anaerobic fermentation occurring in an environment with limited gas exchange. The CMC/GL composite films containing ZLPE may have a certain inhibitory effect on the respiration of cherries. These results demonstrated that the ZLPE-loaded film effectively slowed cherry ripening and delayed the decline of soluble solid content. The above indicators showed that these ZLPE-loaded films have practical significance for fruit preservation.

## 4. Conclusions

In this study, a stable Pickering emulsion containing lemon essential oil was successfully prepared and incorporated into CMC/GL films. CLSM images demonstrated the even dispersal of ZLPE within the film-forming solution. XRD and FT-IR analyses revealed the formation of stronger hydrogen bonds between the emulsion and the film matrix. At lower concentrations, the TS of the films increased with rising ZLPE concentration. Although there was a decreasing trend in TS at 1.0% concentration, it remained higher than that of the control films. The resulting film exhibited superior hydrophobicity and UV-blocking properties compared to the control films. The thermal stability of the ZLPE-loaded film was also slightly improved. Furthermore, the addition of the ZLPE significantly improved the antioxidant capacity and antibacterial activity of the packaging film (*p* < 0.05), increasing the DPPH free radical scavenging rate from 8.17 ± 0.55% to 47.70 ± 2.52% and demonstrating maximum inhibition zone diameters of 12.50 ± 0.56 mm for *E. coli* and 16.70 ± 0.95 mm for *S. aureus*. Ultimately, the ZLPE-loaded film effectively retarded the weight loss, hardness reduction, and decrease in soluble solid content of cherries, suggesting its potential as a food packaging material.

## Data Availability

The original contributions presented in the study are included in the article, further inquiries can be directed to the corresponding authors.
